# Targeted Screening to Predict *Magnusiomyces* Infections in Hematopoietic Cell Transplant Recipients: Evidence from an Outbreak Setting

**DOI:** 10.3390/jof12040254

**Published:** 2026-04-01

**Authors:** Claudia Bartalucci, Chiara Russo, Anna Maria Raiola, Massimiliano Gambella, Vincenzo Di Pilato, Paola Morici, Elena De Carolis, Bram Spruijtenburg, Eelco F. J. Meijer, Monica Melchio, Elisa Balletto, Anna Marchese, Emanuele Angelucci, Matteo Bassetti, Malgorzata Mikulska

**Affiliations:** 1 Division of Infectious Diseases, Department of Health Sciences (DISSAL), University of Genoa, 16132 Genoa, Italy; bartalucciclaudia@gmail.com (C.B.); melchio.monica@gmail.com (M.M.); matteo.bassetti@hsanmartino.it (M.B.); 2 Department of Neuroscience, Rehabilitation, Ophthalmology, Genetics and Maternal and Child Sciences (DiNOGMI), University of Genoa, 16132 Genoa, Italy; chiara.russo16@icloud.com; 3 UOC Ematologia e Terapie Cellulari, IRCCS Azienda Ospedaliera Metropolitana, 16132 Genoa, Italy; annamaria.raiola@hsanmartino.it (A.M.R.); massimiliano.gambella@hsanmartino.it (M.G.); emanuele.angelucci@hsanmartino.it (E.A.); 4 Department of Surgical Sciences and Integrated Diagnostics (DISC), University of Genoa, 16126 Genoa, Italy; vincenzo.dipilato@unige.it (V.D.P.); anna.marchese@unige.it (A.M.); 5 Microbiology Unit, IRCCS Azienda Ospedaliera Metropolitana, 16132 Genoa, Italy; paola.morici@hsanmartino.it; 6 Dipartimento di Scienze di Laboratorio ed Ematologiche, Fondazione Policlinico Universitario “A. Gemelli” IRCCS, 00168 Rome, Italy; elena.decarolis@policlinicogemelli.it; 7 Radboudumc-CWZ Center of Expertise for Mycology, 6532 SZ Nijmegen, The Netherlands; b.spruijtenburg@cwz.nl (B.S.); eelco.meijer@radboudumc.nl (E.F.J.M.); 8 Department of Medical Microbiology and Immunology, Canisius-Wilhelmina Hospital (CWZ)/Dicoon, 6532 SZ Nijmegen, The Netherlands; 9 Infectious Diseases Unit, IRCCS Azienda Ospedaliera Metropolitana, 16132 Genoa, Italy; elisa.balletto@hsanmartino.it

**Keywords:** *Magnusiomyces* spp., rare yeasts, *Geotrichum* spp., *Saprochaete* spp., hematopoietic cell transplantation, systematic screening, fungal outbreak

## Abstract

Invasive infections caused by *Magnusiomyces* spp. are rare, but are associated with severe complications in hematopoietic cell transplantation (HCT) recipients and hospital outbreaks. Following a *Magnusiomyces clavatus* outbreak in our HCT unit, a prospective targeted screening protocol was implemented, which included pharyngeal and rectal swabs cultured on yeast-selective media with prolonged incubation. Clinical and microbiological data were analyzed, and whole-genome sequencing (WGS) was performed on the available isolates. During the study period (September 2022–July 2023), five colonizations and five invasive breakthrough *Magnusiomyces* infections were identified. Despite prompt initiation of antifungal treatment, 4/5 patients (80%) died. WGS demonstrated clonal relatedness among four *M. clavatus* isolates, supporting clonal transmission, although no environmental sources were identified. An enhanced two-phase screening strategy involving 71 patients showed limited benefit, identifying only one additional colonization case compared to routine surveillance cultures. A retrospective review (2007–2021) identified 58 *Magnusiomyces* spp. episodes, with only 10% occurring in patients with hematological malignancies. Our study describes a prolonged clonal outbreak confined to an HCT unit and provides a detailed evaluation of a targeted screening approach in this setting, highlighting the challenges of early identification and prediction of invasive infections. Further studies are needed to define the optimal surveillance and prevention strategies.

## 1. Introduction

Rare yeasts have emerged as uncommon but clinically significant causes of invasive fungal infections (IFIs), particularly in immunocompromised hosts [1]. Among them, *Geotrichum candidum* and *Magnusiomyces* spp. (*M. capitatus*, formerly *Geotrichum capitatum* or *Blastoschizomyces capitatus*, and *M. clavatus*, formerly *Geotrichum clavatum* or *Saprochaete clavata* [2]) have gained attention as opportunistic pathogens responsible for sporadic cases and nosocomial outbreaks, mainly reported in Europe [3,4,5,6,7,8]. These infections most often present as bloodstream infections (BSIs) with possible dissemination to the lungs, liver, central nervous system (CNS), or skin [9,10].

Patients with hematologic malignancies (HM) or those undergoing hematopoietic cell transplantation (HCT) are at the highest risk [11]. Profound and prolonged neutropenia [12,13], severe immunosuppression, intrinsic resistance of *Magnusiomyces* spp. to antifungal prophylaxis agents used in this population (i.e., echinocandins), and high minimum inhibitory concentration (MIC) values for fluconazole are the main predisposing factors [9]. Environmental and hospital reservoirs, including contaminated food and equipment, have also been implicated in outbreaks [14,15].

The diagnosis of *Magnusiomyces* infection relies on conventional microbiological methods, such as cultures, whereas molecular tools can offer complementary information, especially in outbreak settings [10]. Matrix-assisted laser desorption/ionization time-of-flight (MALDI-TOF) can be used for species identification, particularly with updated databases [16], whereas data on the role of fungal biomarkers are limited, with a reported serum β-D-glucan (BDG) sensitivity of 65% [17]. Current guidelines recommend amphotericin B with or without flucytosine or voriconazole for *Magnusiomyces* infections; however the optimal regimen remains undefined due to absent susceptibility breakpoints and a lack of comparative studies [10]. Mortality is high, ranging from 40% to 80% in immunocompromised patients [9,12,18], and no specific recommendations exist for outbreak management, emphasizing the need for early diagnosis, effective treatment, and infection control strategies.

Although several outbreaks of *Magnusiomyces* spp. infections have been reported in patients with HM, to our knowledge, no studies have described structured strategies dedicated to improving patient outcomes during an outbreak. We report the results of a single-center protocol for the detection of *Magnusiomyces* spp. colonization during a HCT outbreak, which we implemented with the aim of early diagnosis and prompt therapy.

## 2. Materials and Methods

### 2.1. Study Design and Patient Population

This monocentric prospective study was conducted at a tertiary care center in Genoa, Italy, with an observation period from September 2022 to July 2023. Following the onset of a *M. clavatus* outbreak in 2022, a targeted screening protocol for colonization was implemented for all HCT recipients from 15 October 2022 to January 2023 and in May 2023. All adult patients (≥18 years) undergoing HCT during this period were systematically screened according to the enhanced protocol described below. For this analysis, all adult HCT recipients with confirmed *Magnusiomyces* spp. infection were included. Moreover, a retrospective analysis (from 2007 to 2021) of cases before the outbreak was conducted to assess the past local epidemiology.

This protocol was developed as a new standard of care risk mitigation strategy to improve outbreak management and was approved by an interdisciplinary committee on infection control convened after the outbreak detection. The data were subsequently analyzed and presented in this study. All patients at our center provided written informed consent for data storage and the use of their clinical data for research purposes at the time of transplantation or cellular therapy procedure and at hospital admissions post-transplant. This study was approved by the Regional Ethics Committee (no. 352/2025) and conducted in accordance with the principles of the Declaration of Helsinki.

### 2.2. Screening Protocol Procedures

At our center, patients receiving HCT routinely undergo colonization screening. It includes a pharyngeal swab to detect bacteria and fungi, a nasal swab to detect filamentous fungi, and a rectal swab to detect carbapenem-resistant *Enterobacterales* (CRE) and vancomycin-resistant enterococci (VRE). Swabs are collected once prior to transplantation, then at least weekly during the post-transplant aplastic phase until engraftment, and subsequently every 1–2 weeks for an additional 3–4 months after engraftment.

Following the detection of an outbreak of *M. clavatus* infections and colonizations in the HCT Unit) in 2022, an internal enhanced screening protocol was introduced in October 2022. This protocol mandates that, in addition to routine screening, all patients hospitalized in the HCT Unit for at least 7–10 days undergo an expanded screening, including an additional pharyngeal swab, rectal swab, and stool sample. The screening sites were selected based on their established role as potential reservoirs for fungal colonization. In the absence of specific evidence regarding the optimal screening strategy for *Magnusiomyces* spp., this approach was partly extrapolated from existing practices for other fungal pathogens and informed by the colonization sites identified through retrospective case analysis at our institution.

The enhanced screening protocol was implemented between 15 October 2022 and 10 January 2023. Following a new case of IFI due to *M. clavatus* in late April 2023, the protocol was reactivated and applied again from 1 May to 31 May 2023. An additional observation period beyond the implemented screening phase was applied until July 2023 to monitor further cases of *Magnusiomyces* infection or colonization. Moreover, an epidemiological investigation aimed at identifying potential common sources of infection was conducted, including environmental sampling of selected areas within the HCT Unit. Samples were collected from patient care environments, including surfaces and shared equipment, as well as from food and water sources, according to local infection control protocols.

### 2.3. Outcomes

The primary aim of this study was to evaluate the effectiveness of enhanced microbiological screening in detecting *Magnusiomyces* spp. colonization and its ability to anticipate the development of invasive infections. The secondary aims included a (i) description of the local epidemiology of *Magnusiomyces* spp. and the (ii) characterization of patients who developed *Magnusiomyces* spp.-related IFI.

### 2.4. Microbiological Procedures

*Magnusiomyces* spp. were identified from clinical and screening samples using MALDI-TOF MS (VITEK MS, bioMérieux, Marcy-l’Etoile, France). Since the change to VITEK MS library v3.2 in 2022, our laboratory has been able to differentiate the three yeasts formerly classified as *Geotrichum* (*G. candidum*, *M. clavatus*, and *M. capitatus*); older libraries allowed for the identification of *M. capitatus* and *G. candidum* only. When MALDI-TOF failed to achieve species-level identification, a microscopic examination was conducted. For cultured isolates, species-level identification was further verified by amplicon sequencing of fungal DNA using the conserved rRNA primers ITS1F and ITS4 [19].

Routine microbiological procedures included: (i) pharyngeal and nasal swabs, seeded on blood and chocolate agar plates, followed by 48 h of incubation at 37 °C; (ii) rectal swabs for the detection of CRE c and VRE, seeded onto selective and differential chromogenic media (Chromatic CRE, Chromatic CRE), followed by 24 h of incubation at 37 °C; (iii) nasal swabs for filamentous fungi, seeded on Sabouraud dextrose agar plates, 7 days of incubation at 30 °C; (iv) bronchoalveolar lavage (BAL) fluid, seeded on blood, chocolate, MacConkey agar plates, and on a chromogenic selective medium for *Candida* spp., followed by 48 h of incubation at 37 °C for bacterial cultures and 7 days at 30 °C for fungal cultures, performed using Sabouraud dextrose agar plates; and (v) blood cultures, followed by 5 days of incubation in an automated system (BACTEC^TM^ Blood Culture System, Becton-Dickinson, Franklin Lakes, NJ, USA). All agar plates were obtained from Liofilchem (Roseto degli Abruzzi, Italy). Given that *Magnusiomyces* spp. typically grow within 3–4 days, enhanced screening consisted of weekly pharyngeal, nasal, and rectal swabs plus stool samples, all seeded on Sabouraud dextrose agar plates and incubated at 30 °C for 7 days.

Antifungal susceptibility testing was performed by broth microdilution (Sensititre YeastOne, Thermo Scientific, Waltham, MA, USA), testing MICs for azoles, echinocandins, and amphotericin B. Serum BDG and galactomannan (GM) results were collected, when available, for patients with invasive infection and colonization and were included in the analysis. GM testing (Platelia^TM^ Aspergillus EIA, Bio-Rad Laboratories, Hercules, CA, USA) was considered positive at an optical density index (ODI) ≥ 0.5 for BAL and serum; values above the detection limit were capped at 10. BDG testing (Fungitell^®^, Associates of Cape Cod, Falmouth, MA, USA) was considered positive at a BDG level of ≥80 pg/mL.

Most of the strains (8/10) of *M. clavatus* and *M. capitatus* causing invasive infections or colonization in HCT recipients (from HSM-S1 to S5 and from HSM-S7 to S9) from September 2022 were analyzed to assess clonal relatedness using whole-genome sequencing (see below). An additional *M. clavatus* strain isolated from a solid organ transplant recipient with IFI was included in the analysis (HSM-S6). This case was not described in the present report because it was unrelated to the outbreak being investigated. Genomic DNA was extracted using the MagNA Pure 96 system and MagNA Pure DNA and Viral Small Volume Kit (Roche Diagnostics, Basel, Switzerland) according to the manufacturer’s instructions. Genomic libraries were prepared using the Nextera DNA Flex kit (lllumina, San Diego, CA, USA) following standard procedures, and paired-end 2 × 150 bp mode sequencing on the Illumina NextSeq 550 system was conducted. Read data were aligned against the *M. clavatus* (GCA_051013725.1) or *M. capitatus* (GCA_900497725.1) reference genome, followed by alignment filtering and single-nucleotide polymorphism (SNP) calling, as previously described in detail [20]. For both species, seven randomly selected isolates from the NCBI SRA database were included as control strains (Table S1). Raw read data generated during the present study were submitted to the NCBI SRA database under BioProject ID PRJNA1347058.

## 3. Results

### 3.1. Outbreak Description

In September 2022, for the first time in our HCT Unit, two patients developed IFI due to *M. clavatus* and died. The first case involved a 70-year-old man with acute myeloid leukemia (AML) in remission (Patient 1) who underwent HCT and later required a second transplant due to graft failure. He developed *M. clavatus* central venous catheter (CVC)-related BSI during prolonged neutropenia while receiving micafungin prophylaxis (+49 days from the first the HCT and +7 days from the second HCT), with radiologically suspected splenic involvement and colonization detected shortly before the onset of infection (negative pharyngeal swabs until day + 47 from the first HCT). GM was consistently negative, whereas BDG was positive 2 days after BSI onset. Despite CVC removal and treatment with liposomal amphotericin B (L-AmB), the patient died a few days later with persistently positive blood cultures. The second case was a 51-year-old man with myelofibrosis (Patient 2), who developed disseminated *M. clavatus* infection with radiologically suspected but not microbiologically documented CNS involvement (Figure S1) 12 days after allogeneic HCT while on micafungin prophylaxis. Also in this case, GM determinations were negative, while BDG was positive 2 days after BSI onset. Despite escalation to combination therapy with L-AmB and voriconazole added few days later, the patient died due to neurological deterioration.

### 3.2. Retrospective Analysis of Cases

Since these were the first *M. clavatus* isolates identified at our center, we retrospectively reviewed all *Magnusiomyces* spp. isolated between 2007 and 2021, including fungi previously classified as *Geotrichum*, *Saprochaete*, or *Magnusiomyces* species. Among 58 patients infected (two cases of BSI) or colonized by *Geotrichum candidum* or *Magnusiomyces* spp., only six (10%) had HM (including one BSI in year 2012 and two colonizations due to *M. capitatus*), while none had *M. clavatus*. The data are summarized in Table S2.

No *M. clavatus* strains were detected before 2022. Retrospective analysis identified the first cases of *M. clavatus* between April and September 2022, when four patients at our hospital were either colonized or infected with the pathogen. The first case was a liver-transplant recipient with disseminated infection transferred from another center, the second was a colonized HCT recipient without progression to invasive fungal disease, and the remaining two were the previously described HCT recipients who developed fatal invasive infections and were considered index cases of the outbreak.

Owing to the temporal and spatial clustering of *M. clavatus* cases observed within the HCT Unit, the hospital infection control team conducted epidemiological investigations to limit further spread and identify a potential common source; however, no environmental origin for the cluster was detected. Consequently, a multidisciplinary meeting was convened, and an internal screening protocol was implemented for all patients admitted to the HCT ward for more than 7 days.

### 3.3. Screening Protocol Implementation (First Phase)

The first phase of enhanced screening was conducted between October 2022 and January 2023, during which 47 patients in the HCT Unit underwent additional screening alongside routine surveillance. Enhanced screening was performed at a mean of 16.4 days after admission. A single timepoint screening was performed in 16 (34%) patients, two in 28 (59.6%) patients, and three weekly screenings in three (6.4%) patients. During this period, enhanced screening identified only one additional case of *M. clavatus* colonization (pharyngeal swab) in a patient who consistently tested negative by routine screening and did not develop an invasive infection. One patient (Patient 5) developed an invasive pulmonary infection due to *M. capitatus* (Figure S2), with fungal isolation from BAL fluid (BALF). Notably, screening performed 19 and 8 days prior to BALF sampling was negative; however, both enhanced and routine screening performed after the BAL procedure were positive for *M. capitatus*. The patient, a 75-year-old man with relapsed/refractory non-Hodgkin lymphoma undergoing Chimeric Antigen Receptor T-cell therapy (CAR-T), died 62 days post-infusion. While fungal infection likely contributed to the patients’ death; grade IV cytokine release syndrome (CRS) was the reason for Intensive Care Unit (ICU) transfer and the primary cause of death. Based on these findings, which show the limited benefit of enhanced screening beyond routine surveillance, the strategy was discontinued.

### 3.4. Screening Protocol Implementation (Second Phase)

However, following a new case of *M. clavatus* IFI diagnosed on 26 April 2023, the enhanced screening program was reinstated throughout May 2023. The case involved a 65-year-old man (Patient 3) with AML in complete remission who underwent allogeneic HCT and developed severe graft-versus-host disease (GvHD). He developed *M. clavatus* BSI on day + 51 post-transplant, successfully treated with 17 days course of L-AmB, although a breakthrough infection occurred on day + 78, and the patient died on day + 91 with negative blood cultures.

In the second phase (from 1 May to 31 May 2023), 24 patients underwent enhanced screening, performed at a mean of 21.8 days after admission to HCT unit. A single timepoint samples were collected from seven (29.2%) patients and two timepoints samples from 17 (70.8%) patients. No new colonizations by *Magnusiomyces* spp. were identified; the single positive sample confirmed colonization in a patient with an already known case of *M. clavatus* IFI.

An additional case of IFI was observed after the second phase of enhanced screening (in July 2023) in a 55-year-old woman with myelofibrosis (Patient 4). The isolate from the blood culture initially identified *Blastoschizomyces capitatus* using MALDI-TOF. Subsequent identification using updated MALDI-TOF libraries indicated the presence of both *M. capitatus* and *M. clavatus*, and WGS ultimately confirmed *M. clavatus*.

**Figure 1:**
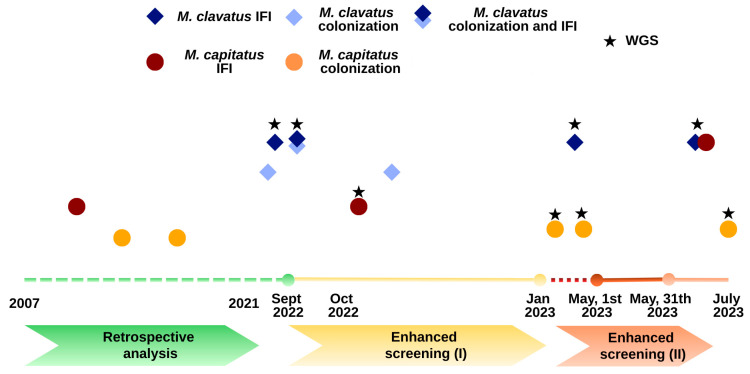
Cases of *Magnusiomyces* spp. colonization and/or infection in patients with hematological malignancies diagnosed at our hospital between 2007 and 2023. Abbreviations: IFI: invasive fungal infections; WGS: whole-genome sequencing.

All strains of *Magnusiomyces* spp. isolated in our hospital from 2007 to 2023 are presented in Figure 1 for patients with HM and Figure S3 for all patients.

### 3.5. Patient Characteristics and Microbiological Findings During Screening Protocol

The baseline characteristics and screening results of the 71 patients who underwent the enhanced screening protocol across both periods are presented in Table 1. Most patients (49.3%) underwent allogeneic HCT, 43.7% received autologous HCT, and 5.6% underwent CAR-T therapy. Colonization with *Magnusiomyces* spp. was detected in two patients on routine screening cultures and in three patients during enhanced screening. The implemented enhanced screening allowed for the identification of only one case of *M. clavatus* colonization in addition to regular screening and other culture examinations. Follow-up screening data were available for only two out of the five colonized patients, showing a duration of colonization of 13 and 15 days, respectively. The clinical characteristics of the colonized patients are presented in Table S3.

Colonization with other rare fungi (*Saccharomyces* spp., n = 5, *Penicillium* spp. n = 5, *Rhodotorula* spp. n = 3, *Cryptococcus laurentii* n = 1) was more frequently detected with enhanced screening (18.3% vs. 1.4%). However, none of these cases was associated with IFI.

### 3.6. Molecular Characterization of Magnusiomyces spp. Strains

Among patients with HM, in the period of 2022 and 2023, there were five cases of IFI and five cases of colonization by *Magnusiomyces* spp. (Table 2, Table 3 and Table S3).

WGS SNP phylogenetic analysis was performed in all five cases of invasive infection and in three out of five colonization cases.

**Figure 2:**
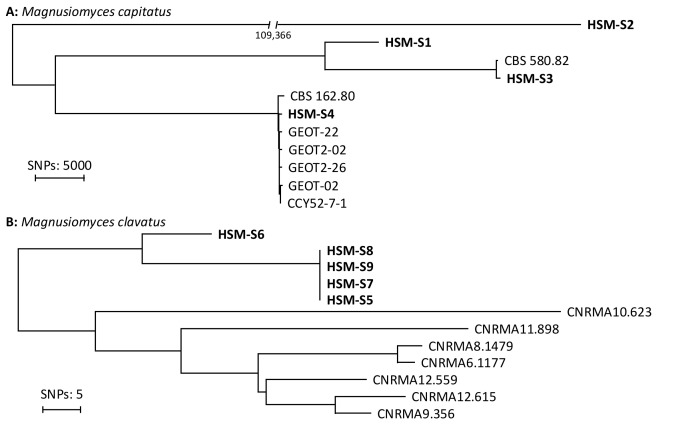
Whole-genome sequencing (WGS) single-nucleotide polymorphism (SNP) phylogenetic analysis of *Magnusiomyces capitatus* (**A**) and *Magnusiomyces clavatus* (**B**). Isolates in bold were collected during the current study, and the number below the branch indicates the SNP difference. Other strains are controls from different centers (see Table S1). Timing of isolation for each patient: HSM-S2 (March 2023), HSM-S1 (March 2023), HSM-S3 (October 2023), HSM-S4 (November 2022), HSM-S6 (July 2022, solid organ transplant recipient not included in the present analysis), HSM-S8 (September 2022), HSM-S9 (April 2023), HSM-S7 (September 2022), and HSM-S5 (July 2023). Please refer to Table 3 and Table S3 for complete information on patients with invasive infection and colonization, respectively. All isolates included in the WGS analysis are shown in Figure 1, illustrating the temporal distribution of isolates.

The WGS SNP analysis showed that all four strains from patients with *M. clavatus* invasive infections occurring in the HCT ward were genetically identical (median separating SNP: 0), supporting clonal expansion (Figure 2). In contrast, WGS analysis demonstrated a high genetic distance of at least 15,000 SNPs between different *M. capitatus* strains and between *M. clavatus* identified in SOT recipients outside HCT outbreak (HSM-S6). In Table S4, we report the MIC values of the five strains that caused IFI. For one patient, antifungal susceptibility testing (AFST) was performed on two samples, since, during the second time, he developed a breakthrough infection while still on L-AmB therapy, with no increase in MIC values.

### 3.7. Clinical Outcomes

The overall mortality among colonized and infected patients was 60% (6/10). Despite all five patients with IFI starting promptly antifungal therapy (in mean 2.2 days after the diagnosis, range 1–4), four out of five (80%) died shortly after the onset of infection. In three out of four deceased patients, other factors significantly contributed to death (GvHD, persistent neutropenia, and CRS), while in one case, *Magnusiomyces* infection was considered the primary cause of death in the early post-engraftment period (Table 3). Among colonized patients, two out of five (40%) died. Both were admitted to the ICU and colonized at the respiratory level.

After the study period, in 2025, an additional case of invasive infection (BSI) due to *M. clavatus* occurred in a transplant recipient hospitalized in the same HCT unit. Preliminary clonal characterization by Fourier transform infrared (FTIR) spectroscopy showed a high relatedness to *M. clavatus* isolates previously recovered in the unit, and the clinical outcome was unfavorable.

## 4. Discussion

The main findings of this study are lack of clear benefit on the clinical impact of an enhanced screening protocol for *Magnusiomyces* during an outbreak caused *M. clavatus* in an HCT unit and the very high clonal relatedness of the strains isolated over a prolonged time period, suggesting a possible environmental reservoir.

To our knowledge, this is the first real-life application of a structured prospective screening approach tailored to *Magnusiomyces* spp. in immunocompromised patients, a field where standardized protocols and evidence are currently lacking. In our experience, the protocol did not allow to detect cases of colonization preceding development of IFI, but the overall number of cases was limited, with long intervals between some of them. More extensive or more frequent screening might have been necessary, since colonization was detected at the time of infection in two out of five cases.

The occurrence of two fatal *M. clavatus* infections in HCT recipients in 2022 raised concern for a potential outbreak, which was subsequently confirmed by WGS, which has been increasingly recognized as a key tool in the investigation of *Magnusiomyces* outbreaks, allowing for the high-resolution assessment of clonal relatedness [5].

This species had not been previously reported in our hospital, likely due to its absence from earlier MALDI-TOF databases (i.e., older than v.3.2 for the Vitek MS platform). A retrospective search for *Magnusiomyces* cases (including all past names) identified no additional cases in the preceding ten years. Notably, WGS analysis showed that *M. capitatus* infections and colonizations in HCT recipients were not linked to clonal transmission, suggesting independent acquisition from unrelated sources. In contrast, *M. clavatus* cases shared a common source, likely within the HCT unit. These findings highlight the role of genomic surveillance in rapidly distinguishing transmission patterns and guiding infection control strategies.

Recently, more clonal outbreaks by *M. clavatus* have been reported in Europe, and the source often remains undetected, as in this study [7]. Given the need to proceed with HCT activity and the high mortality associated with *Magnusiomyces* spp. infections [9,12], a general audit of infection control policies was performed and a targeted screening protocol was developed and implemented. The aim was to determine whether a strategy of close surveillance could allow us to promptly detect *Magnusiomyces* colonization and enable targeted prophylaxis or early therapy. However, despite its application in 71 HCT recipients across two distinct timeframes, the enhanced protocol identified only one additional case of *M. clavatus* colonization beyond those detected by routine surveillance cultures. Notably, all other colonizations and infections were either identified by standard pharyngeal swabs (growing on standard culture media within 48 h) or through clinically driven sampling (e.g., BAL or blood cultures), and detection of colonization occurred very close to the onset of IFI, suggesting that the diagnostic window for early detection may be narrow or absent. Unlike *Candida* spp. or *Aspergillus* spp., for which colonization is often a prelude to invasive disease [21], the transition from colonization to infection for *Magnusiomyces* spp. appears to occur rapidly, possibly reflecting a sudden increase in fungal burden and very severe host immune suppression. Among the five patients with colonization alone, none subsequently developed invasive disease, including the three patients alive at the last follow-up and the two who died from unrelated causes. In two patients with available follow-up testing for colonization, colonization appeared to be a transient event, with a mean duration of 14 days.

Screening performance may have been influenced by the predefined selection of sites. Skin sites were not included, since they were rarely reported as positive in previous reports (Table S2). Moreover, we have never detected *Magnusiomyces* spp. growing in *Candida auris* screening samples (bilateral axilla and groin swabs) at our hospital, despite an extensive *C. auris* screening program.

Our findings highlight the unclear clinical significance of *Magnusiomyces* spp. colonization. Nevertheless, the detection of *Magnusiomyces* spp. in any sample should be considered a warning sign in high-risk patients, warranting careful clinical evaluation. Very recently, an additional case of urinary colonisation with *M. clavatus* in a HCT recipient was followed by blodstream infection (case not included in this study). Larger studies are needed to better define the kinetics and clinical impact of colonization by these pathogens.

From a non-culture based diagnostic standpoint, our study confirms the lack of utility of GM in detecting *Magnusiomyces* spp. infections, since all cases tested negative, as observed in other reports [10]. Conversely, serum BDG was consistently elevated in patients with confirmed IFI; however, positivity was generally observed after infection onset, with only one case showing earlier positivity (23 days before). In two cases, pre-infection BDG measurements were not available, as BDG screening is used in our center only in case of high-risk patients who are not on echinocandin prophylaxis. Thus, BDG might contribute to early suspicion of this IFI if used as general or targeted screening.

An interesting finding of our study was the detection, in a single patient (Patient 4), of both *M. clavatus* and *M. capitatus* in blood cultures based on MALDI-TOF identification, a finding not previously reported in the literature. Polymicrobial invasive infections involving *Saprochaete*, *Magnusiomyces*, or *Geotrichum* species have not been described in the largest available cohorts [9,12], and published cases consistently report monomicrobial disease; however, *M. clavatus* and *M. capitatus* are phylogenetically closely related species and may be easily misidentified by MALDI-TOF MS. In our case, the initial identification as *Blastoschizomyces capitatus* (currently *M. capitatus*) was likely influenced by the predominance of one species within the clinical sample, as also supported by the WGS-based identification that exclusively yielded *M. clavatus*. The presence of the second colony morphotype was indeed observed only after subculturing of the original isolates for investigational purposes, consistent with our hypothesis, and subsequent MALDI-TOF analysis yielded results consistent with a mixed infection. This finding highlights the limitations of MALDI-TOF MS systems in differentiating phylogenetically related taxa, particularly when reference spectral databases are incomplete or not regularly updated, and the risk that mixed infections may be missed within routine diagnostic workflows when one species predominates in the clinical specimen.

A worrisome aspect of this study is the alarmingly high mortality associated with *Magnusiomyces* spp. infections, with four out of five patients (80%) dying despite prompt antifungal therapy (median time from diagnosis to treatment initiation: 2 days). This is consistent with prior reports that suggest poor outcomes even with L-AmB or combination regimens [9,12,18]. 

Bloodstream infection was the predominant clinical presentation in our series, consistent with previous reports [9,12]. The duration of positive blood cultures was relatively prolonged in 2/4 patients (7 and 8 days). One patient died with persistently positive blood cultures, while another died with the first negative set obtained on the day of death. A third patient experienced infection relapse one month later, after a 17-day course of therapy and documented blood culture clearance. No deep-seated focus of infection was identified, and culture of the CVC removed after the second episode was negative, leaving the mechanism of relapse unclear. Three patients also showed deep-organ dissemination, with abdominal involvement in two cases and the much rarer cerebral localization in the other. These were suspected based on radiological findings in the absence of other plausible pathogens, but without microbiological confirmation from deep sampling due to trombocytopoenia. Cerebral involvement has been rarely reported in literature (only 2 and 23 cases in the FungiScope registry [12]) and represents a major diagnostic and management challenge, often fatal [10], as in our case.

The patients in our series received antifungal initial monotherapy (L-AmB in 4 and voriconazole in 1), with addition of azoles to L-AmB after a few days in two cases, as current guidelines provide no clear recommendations on combination therapy due to limited evidence [10]. However, the high mortality and the challenges posed by difficult sites such as CNS involvement should be considered in treatment decisions. An important factor independent of therapy is neutrophil recovery. In our series, the only surviving patient had documented neutrophil recovery at the time of infection and the shortest duration of bloodstream infection (3 days), despite splenic and hepatic involvement, whereas most fatal cases were characterized by profound and prolonged neutropenia, confirming its key role as a determinant of outcome [9].

The strengths of our study include the integration of a structured protocol into clinical practice, prospective data collection during an active outbreak, and detailed microbiological analysis. The limitations of this study include the small number of colonization and invasive infection cases, which prevents drawing robust conclusions about the effectiveness of the screening strategy. Given the rarity of *Magnusiomyces* spp. infections, studies in this field are inevitably affected by limited sample sizes. Moreover, the screening strategy was partially extrapolated from surveillance approaches used for other fungal pathogens, as no data on colonization with *Magnusiomyces* spp. in neutropenic patients was available. Finally, despite testing, no environmental source was identified, preventing a comprehensive investigation of transmission dynamics.

## 5. Conclusions

This study highlights the challenges in early identification of *Magnusiomyces* spp. colonization through intensive screening during an outbreak, possibly reflecting a narrow window between colonization and invasive disease. In this context, screening may have limited predictive value for anticipating invasive infection; however, these findings should be interpreted with caution, given the small sample size. Nevertheless, the high mortality associated with *Magnusiomyces* infections underscores the importance of heightened clinical vigilance and prompt diagnostic and therapeutic interventions in high-risk HCT recipients. Further multicenter studies, ideally incorporating molecular epidemiology and environmental surveillance, are warranted to better define the role of screening and pre-emptive strategies in the management of these rare but devastating yeast infections.

## Figures and Tables

**Table 1:** Baseline characteristics of screened patients.

Baseline Characteristics	N (%)
**Total patients screened**	71
**Mean age (min–max)**	58 (25–75)
**Sex**	
Female	24 (33.8)
Male	47 (66.2)
**Type of cellular therapy**	
Allogeneic	35 (49.3)
Autologous	31 (43.7)
CAR-T	4 (5.6)
HCT candidate	1 (1.4)
**Mean time from HCT-unit admission to standard and implemented screening, days (min–max)**	19.9 (3–122)
***Magnusiomyces*** **spp. colonization**	
Regular screening/culture	2 (2.8) *
Implemented screening	3 (4.2) *
**Other rare yeasts colonization**	
Regular screening/culture	1 (1.4%)
Implemented screening	13 (18.3)
***Candida*** **spp. colonization**	
Regular screening/culture	5 (7%)
Implemented screening	20 (28.2)
**IFI**	2 (2.8%) *

Abbreviations: HCT: hematopoietic stem cell transplantation; IFI: invasive fungal disease; * two cases of IFI due to *M. capitatus* and *M. clavatus* (formerly *G. capitatum* and *S. clavata*) in which the isolation in bronchoalveolar lavage fluid and blood, respectively, preceded the isolation by the implemented screening.

**Table 2:** Characteristics of patients infected or colonized by *Magnusiomyces* spp. in 2022 and 2023.

Baseline Characteristics	IFI (N = 5)	Colonization (N = 5)
**Mean age (min–max)**	63.2 (55–75)	65.6 (57–78)
**Sex, n (%)**		
Female	1 (20)	1 (20)
Male	4 (80)	4 (80)
**Hematological disease, n (%)**		
AML	2 (40)	3 (60)
NHL	1 (20)	1 (20)
Myelofibrosis	2 (40)	0 (0)
Multiple myeloma	0 (0)	1 (20)
**Type of cellular therapy, n (%)**		
Allogenic	4 (80)	3 (60)
Autologous	0 (0)	2 (40)
CAR-T	1 (20)	0 (0)
**Admission ward, n (%)**		
HCT Unit	5 (100)	4 (80)
Other	0 (0)	1 (20)
**Mean time from HCT to IFI/colonization, days (min–max)**	37 (12–51)	45 (15–127 *)
**Neutropenia at time of IFI/colonization isolation, n (%)**		
Yes	3 (60)	2 (40)
No	2 (40)	3 (60)
**Mean neutropenia duration before IFI/colonization, days (min–max)**	25.3 (10–47)	32.5 (15–50)
**Microbiological sample, n (%)**		
Blood	4 (80)	0 (0)
BALF/BAS	1 (20)	2 (40)
Pharyngeal swab	0 (0)	2 (40)
**Rectal swab (implemented screening)**	0 (0)	1 (20)
***Magnusiomyces*** **spp., n (%)**		
*M. clavatum*	3 (60)	2 (40)
*M. capitatum*	1 (20)	3 (60)
Mixed infection: *M. capitatus* and *M. clavatus*	1 (20)	0 (0)
**BDG positive, n (%)**		
Yes	5 (100)	0 (0)
No	0 (0)	5 (100)
**GM positive, n (%)**		
Yes	1 (20)	0 (0)
No	4 (80)	5 (100)
**Antifungal prophylaxis at time of IFI/colonization, n (%)**		
Yes	5 (100); MFG	4/ (80); 3 MFG, 1 POS
No	0 (0)	1 (20)
**Mean time from IFI to antifungal therapy, days (min-max)**	2.2 (1–4)	NA
**30-day all-cause mortality**		
Deceased	4 (80)	2 (40)
Alive	1 (20)	3 (60)

Abbreviations: AML: acute myeloid leukemia; BDG: beta-D-glucan; GM: galactomannan; HCT: hematopoietic stem cell transplantation; IFI: invasive fungal infection; MFG: micafungin; NHL: non-Hodgkin lymphoma; POS, posaconazole. * One patient who underwent autologous HCT 8 years before BALF isolation was excluded. He was already colonized in the sputum in 2015.

**Table 3:** Characteristics of patients with invasive fungal disease caused by *Magnusiomyces* spp.

	*M. clavatus*			Mixed Infection: *M. capitatus* and *M. clavatus*	*M. capitatus*
	Patient 1 (HSM-S7)	Patient 2 (HSM-S8)	Patient 3 (HSM-S9)	Patient 4 (HSM-S5)	Patient 5 (HSM-S4)
Sex,	M	M	M	F	M
Age, years	70	51	65	55	75
Hematological malignancy	AML—complete remission	Myelofibrosis	AML—complete remission	Myelofibrosis	NHL
Type of cellular therapy	Haploidentical HCT; second haploidentical HCT on day + 42 due to graft failure	MUD HCT	Haploidentical HCT	MUD HCT	CAR-T
GvHD	No	Cutaneous grade I, day + 8	Intestinal grade IV, day + 46	No	NA
Neutrophils engraftment, days post-HCT	NA	NA	+18	+16	NA
Comorbidities	HIV (suppressed viral load), ex-smoker	Psoriasis (no systemic drugs)	Diabetes mellitus	Hashimoto’s thyroiditis	Diabetes mellitus, diverticulosis
Days from hospitalization to *Magnusiomyces* spp. isolation	56	20	58	30	71
Days from HCT/CAR-T to *Magnusiomyces* spp. Invasive infection	+49	+12	+51	+22	+50
Days from HCT/CAR-T to *Magnusiomyces* spp. colonization and site	+51; pharyngeal swab	Not colonized	Not colonized	Not colonized	+56; pharyngeal swab
Length of neutropenia (ANC < 500/mmc) at time of *Magnusiomyces* spp. isolation, days	47	10	0	0	19
Antifungal prophylaxis	Micafungin	Micafungin	Micafungin	Micafungin	Micafungin
Microbiological samples positive for *Magnusiomyces* spp.	Blood (from day + 49 to day + 56, still positive at death); pharyngeal swab (day + 55)	Blood (from day + 12 to day + 20, first negative on the day of death)	Blood (from day + 51 to day + 54; again positive on day + 78)	Blood (day + 22, subsequent negative after 3 days)	BALF (day + 50, follow-up BAL not available)
BDG	>523 pg/mL (2 days after BSI; determination not available before BSI)	>523 pg/mL (2 days after BSI; negative 28 days before BSI)	First positive 23 days before BSI: 204 pg/mL, then positive until death	>523 pg/mL (6 days after BSI; determination not available before BSI)	>523 pg/mL (2 days after IFI; negative 9 days before IFI)
GM	Negative	Negative	Negative	Negative	Positive in BALF and in serum (2 days after IFI; negative 9 days before IFI)
IFI localization	CR-BSI with suspected splenic localization	BSI with CNS involvement (Figure S1)	BSI	BSI with splenic and liver localization (Figure S4)	Lung involvement (Figure S2)
Antifungal therapy	L-AmB (started after 2 days)	L-AmB (started after 2 days) plus voriconazole (added after 5 days)	L-AmB (started after 2 days): anidulafungin (started after 4 days) plus isavuconazole (added after 3 days)	L-AmB (started after 4 days); after 18 days, switch to voriconazole; after another 23 days, switch to isavuconazole	Voriconazole (started after 1 day)
Outcome (cause of death)	Death (*Magnusiomyces* infection in prolonged neutropenia)	Death (*Magnusiomyces* infection in recent engraftment)	Death (Hematological disease complication, blood culture negative for *Magnusiomyces* at the time of death)	Alive	Death (CRS grade IV)

Abbreviations: ANC: absolute neutrophil count; AML: acute myeloid leukemia; BDG: beta-D-glucan; CAR-T: Chimeric Antigen Receptor T-cell therapies; CNS: central nervous system; CR-BSI: catheter related bloodstream infection; CRS: Cytokine Release Syndrome; F: female; GM: galactomannan; GvHD: Graft versus Host Disease; HCT: hematopoietic stem cell transplantation; L-AmB: liposomal amphotericin B; M: male; MUD: matched unrelated donor; NA: non-available; NHL: non-Hodgkin lymphoma.

## Data Availability

The original contributions presented in this study are included in the article/Supplementary Material. Further inquiries can be directed to the corresponding author.

## Supplementary Materials

The following supporting information can be downloaded at https://www.mdpi.com/article/10.3390/jof12040254/s1. Table S1: *Magnusiomyces* spp. control isolates included in the whole-genome sequencing (WGS) single-nucleotide polymorphism (SNP) analysis; Table S2: *Magnusiomyces* spp. isolated at our hospital from 2007 to 2021 in hematological or non-hematological patients; Table S3: Characteristics of patients colonized by *Magnusiomyces* spp.; Table S4: Available antifungal Minimum Inhibitory Concentration of *M. clavatus* and *M. capitatus* in patients with IFI, 2022 and 2023; Figure S1: Magnetic Resonance Imaging of CNS disease due to *M. capitatus*; Figure S2: Lung CT Imaging of pulmonary disease due to *M. capitatus*; Figure S3: *Geotrichum* spp. and *Magnusiomyces* spp. strains isolated in our hospital from 2007 to 2023 with regular screening and routine microbiological tests; Figure S4: Hepatosplenic invasive fungal disease due to *M. clavatus*.

## References

[1] Kontoyiannis D.P., Marr K.A., Park B.J., Alexander B.D., Anaissie E.J., Walsh T.J., Ito J., Andes D.R., Baddley J.W., Brown J.M. (2010). Prospective Surveillance for Invasive Fungal Infections in Hematopoietic Stem Cell Transplant Recipients, 2001–2006: Overview of the Transplant-Associated Infection Surveillance Network (TRANSNET) Database. Clin. Infect. Dis..

[2] Kidd S.E., Abdolrasouli A., Hagen F. (2023). Fungal Nomenclature: Managing Change Is the Name of the Game. Open Forum Infect. Dis..

[3] Gurgui M., Sanchez F., March F., Lopez-Contreras J., Martino R., Cotura A., Galvez M.L., Roig C., Coll P. (2011). Nosocomial Outbreak of Blastoschizomyces Capitatus Associated with Contaminated Milk in a Haematological Unit. J. Hosp. Infect..

[4] Del Principe M.I., Sarmati L., Cefalo M., Fontana C., De Santis G., Buccisano F., Maurillo L., De Bellis E., Postorino M., Sconocchia G. (2016). A Cluster of Geotrichum Clavatum (Saprochaete Clavata) Infection in Haematological Patients: A First Italian Report and Review of Literature. Mycoses.

[5] Lo Cascio G., Vincenzi M., Soldani F., De Carolis E., Maccacaro L., Sorrentino A., Nadali G., Cesaro S., Sommavilla M., Niero V. (2020). Outbreak of Saprochaete Clavata Sepsis in Hematology Patients: Combined Use of MALDI-TOF and Sequencing Strategy to Identify and Correlate the Episodes. Front. Microbiol..

[6] García-Ruiz J.C., López-Soria L., Olazábal I., Amutio E., Arrieta-Aguirre I., Velasco-Benito V., Pontón J., Moragues M.-D. (2013). Invasive Infections Caused by Saprochaete Capitata in Patients with Haematological Malignancies: Report of Five Cases and Review of the Antifungal Therapy. Rev. Iberoam. Micol..

[7] Vaux S., Criscuolo A., Desnos-Ollivier M., Diancourt L., Tarnaud C., Vandenbogaert M., Brisse S., Coignard B., Dromer F., The Geotrichum Investigation Group (2014). Multicenter Outbreak of Infections by Saprochaete Clavata, an Unrecognized Opportunistic Fungal Pathogen. mBio.

[8] Stanzani M., Cricca M., Sassi C., Sutto E., De Cicco G., Bonifazi F., Bertuzzi C., Bacci F., Paolini S., Cavo M. (2019). Saprochaete Clavata Infections in Patients Undergoing Treatment for Haematological Malignancies: A Report of a Monocentric Outbreak and Review of the Literature. Mycoses.

[9] Del Principe M.I., Seidel D., Criscuolo M., Dargenio M., Rácil Z., Piedimonte M., Marchesi F., Nadali G., Koehler P., Fracchiolla N. (2023). Clinical Features and Prognostic Factors of Magnusiomyces (Saprochaete) Infections in Haematology. A Multicentre Study of SEIFEM/Fungiscope. Mycoses.

[10] Chen S.C.-A., Perfect J., Colombo A.L., Cornely O.A., Groll A.H., Seidel D., Albus K., De Almedia J.N., Garcia-Effron G., Gilroy N. (2021). Global Guideline for the Diagnosis and Management of Rare Yeast Infections: An Initiative of the ECMM in Cooperation with ISHAM and ASM. Lancet Infect. Dis..

[11] Fernández-Ruiz M., Guinea J., Puig-Asensio M., Zaragoza Ó., Almirante B., Cuenca-Estrella M., Aguado J.M., on behalf of the CANDIPOP Project, GEIH-GEMICOMED (SEIMC) and REIPI (2017). Fungemia Due to Rare Opportunistic Yeasts: Data from a Population-Based Surveillance in Spain. Med. Mycol..

[12] Durán Graeff L., Seidel D., Vehreschild M.J.G.T., Hamprecht A., Kindo A., Racil Z., Demeter J., De Hoog S., Aurbach U., Ziegler M. (2017). Invasive Infections Due to Saprochaete and Geotrichum Species: Report of 23 Cases from the FungiScope Registry. Mycoses.

[13] Girmenia C., Pagano L., Martino B., D’Antonio D., Fanci R., Specchia G., Melillo L., Buelli M., Pizzarelli G., Venditti M. (2005). Invasive Infections Caused by Trichosporon Species and Geotrichum Capitatum in Patients with Hematological Malignancies: A Retrospective Multicenter Study from Italy and Review of the Literature. J. Clin. Microbiol..

[14] Pottier I., Gente S., Vernoux J., Gueguen M. (2008). Safety Assessment of Dairy Microorganisms: Geotrichum Candidum. Int. J. Food Microbiol..

[15] Menu E., Criscuolo A., Desnos-Ollivier M., Cassagne C., D’Incan E., Furst S., Ranque S., Berger P., Dromer F. (2020). Saprochaete Clavata Outbreak Infecting Cancer Center through Dishwasher. Emerg. Infect. Dis..

[16] Kolecka A., Khayhan K., Groenewald M., Theelen B., Arabatzis M., Velegraki A., Kostrzewa M., Mares M., Taj-Aldeen S.J., Boekhout T. (2013). Identification of Medically Relevant Species of Arthroconidial Yeasts by Use of Matrix-Assisted Laser Desorption Ionization–Time of Flight Mass Spectrometry. J. Clin. Microbiol..

[17] Forster J., Koc Ö., Koeppel M.B., Hamprecht A., Kurzai O., Suerbaum S., Wagener J., Dichtl K. (2022). β-1,3-d-Glucan and Galactomannan as Biomarkers for the Detection of Invasive Geotrichum and Magnusiomyces Infections: A Retrospective Evaluation. J. Clin. Microbiol..

[18] El Zein S., Hindy J.-R., Kanj S.S. (2020). Invasive Saprochaete Infections: An Emerging Threat to Immunocompromised Patients. Pathogens.

[19] White J., Bruns T., Lee S., Taylor J. (1990). Amplification and Direct Sequencing of Fungal Ribosomal RNA Genes for Phylogenetics. PCR—Protocols and Applications—A Laboratory Manual.

[20] Thakur S., Spruijtenburg B., Abhishek, Shaw D., De Groot T., Meijer E.F.J., Narang T., Dogra S., Chakrabarti A., Meis J.F. (2025). Whole Genome Sequence Analysis of Terbinafine Resistant and Susceptible Trichophyton Isolates from Human and Animal Origin. Mycopathologia.

[21] Nucci M., Anaissie E. (2014). How We Treat Invasive Fungal Diseases in Patients with Acute Leukemia: The Importance of an Individualized Approach. Blood.

