# Supplementary material for: Targeted Screening to Predict Magnusiomyces Infections in Hematopoietic Cell Transplant Recipients: Evidence from an Outbreak Setting
Source: J Fungi (Basel). 2026 Apr 1;12(4):254. doi: 10.3390/jof12040254 (PMC13117050; doi:10.3390/jof12040254)

Supplementary Materials

TABLES

Table S1. *Magnusiomyces* spp. control isolates included in the whole genome sequencing (WGS) single nucleotide polymorphism (SNP) analysis.

ID	Species	SRA accession
CBS 162.80	*M. capitatus*	ERR2896329
GEOT-22	*M. capitatus*	ERR2752241
GEOT2-02	*M. capitatus*	ERR2752252
GEOT2-26	*M. capitatus*	ERR2752253
GEOT-02	*M. capitatus*	ERR2752208
CCY52-7-1	*M. capitatus*	ERR2752259
CBS 580.82	*M. capitatus*	ERR2752133
CNRMA6.1177	*M. clavatus*	ERR339474
CNRMA10.623	*M. clavatus*	ERR339468
CNRMA11.898	*M. clavatus*	ERR339461
CNRMA12.615	*M. clavatus*	ERR339471
CNRMA9.356	*M. clavatus*	ERR339476
CNRMA12.559	*M. clavatus*	ERR339470
CNRMA8.1479	*M. clavatus*	ERR339467

Table S2. *Magnusiomyces* spp. isolated at our hospital from 2007 to 2021 in hematological or non-hematological patients.

No cases of *M. clavatus*, formerly (*Saprochaete clavata*) were identified.

		Blastoschizomyces capitatus*	***Geotrichum candidum***	***Geotrichum spp.****	**Total in HM**	**Total *n***
		n.; Microbiological sample	*n.;* Microbiological sample	*n.;* Microbiological sample		
2021	**HM**				**0**	5
	non-HM	5; 2 sputum, 1 BAS, 1 wound swab, 1 pharyngeal swab				
**2020**	**HM**				**0**	1
	non-HM		1; 1 drainage fluid			
2019	**HM**				**0**	5
	non-HM	3; 2 sputum, 1 UC	2; 1 sputum, 1 stool			
2018	**HM**	**1; pharyngeal swab**			**1**	8
	non-HM	5; 1 blood, 1 BALF, 2 BAS, 1 UC	2; 1 BALF, 1 BAS			
2017	**HM**				**0**	2
	non-HM	1; vaginal swab		1; abdominal fluid		
2016	HM				**0**	3
	non-HM		3; 2 BAS, 1 pharyngeal swab			
2015	**HM**	**1; sputum**			**1**	4
	non-HM	3; 1 BAS, 2 sputum				
2014	**HM**				**0**	3
	non-HM	1; pharyngeal swab	1; BALF	1; BAS		
2013	**HM**				**0**	4
	non-HM	4; 1 BALF, 3 sputum				
2012	**HM**	**1; blood**			**1**	5
	non-HM	3; 1 drainage fluid, 1 BAS, 1 sputum		1; purulent wound		
2011	**HM**				**0**	1
	non-HM	1; sputum				
2010	**HM**			1; pharyngeal swab	**1**	4
	non-HM	3; 1 BALF, 1 BAS, 1 sputum				
2009	**HM**		1; pharyngeal swab	1; pharyngeal swab	**2**	10
	non-HM	6; 3 BAS, 1 sputum, 2 pharyngeal swab	2; 1 sputum, 1 BAS			
2008	**HM**				**0**	3
	non-HM	3; 1 BALF, 1 BAS, 1 wound swab				
2007	**HM**				**0**	0
	non-HM					

Abbreviations: BALF: bronchoalveolar lavage fluid; BAS: bronchoaspirate; HM: hematological malignancy; non-HM: non-hematological malignancy; UC: urine culture. *Species identification is reported according to the nomenclature in use at the time of diagnosis.

Table S3. Characteristics of patients colonized by *Magnusiomyces* spp.

	M. clavatus		***M. capitatus***		
	**Patient 1**	**Patient 2**	**Patient 3 (HSM-S1)**	**Patient 4 (HSM-S2)**	**Patient 5 (HSM-S3)**
Sex	M	M	F	M	M
Age, years	59	57	69	65	78
Hematological disease	AML – complete remission	NHL – complete remission	AML	AML – complete remission after II haploidentical-HCT	MM, HCT autologous in 2015
Type of cellular therapy	MUD HCT	Autologous HCT	MUD HCT	Haploidentical HCT	Autologous HCT
GvHD	Cutaneous, grade I, day +24	NA	Oral mucositis, grade I, day +8	Absent	NA
Neutrophil engraftment, days post-HCT	+24	+10	+15	+28	NA
Comorbidities	HCV-related liver disease, active smoker, arrythmia	Active smoker	Hypertension	Gilbert syndrome	Renal cancer, squamous cell carcinoma
Days of hospitalization at time of *Magnusiomyces* isolation	26	29	28	1	41, already colonized in 2015 (sputum)
Days post-HCT at time of *Magnusiomyces spp.* isolation	+15 and +22	+ 18	+19	+127	NA
Duration of neutropenia (ANC < 500/mmc) at time of isolation, days	15	0, engraftment already occurred	0, engraftment already occurred	50	0
Antifungal prophylaxis	Micafungin	Micafungin	Micafungin	Posaconazole	None
Microbiological sample	Pharyngeal swab	Rectal swab	Pharyngeal swab	BAS	BALF
BDG	Positive (192 pg/mL) one month later	NA	NA	Negative 8 days before	Positive (96 pg/mL) 18 days before, then negative
GM	Negative	Negative	Negative	Negative	NA
Outcome<br>Cause of death	Alive	Alive	Alive	Death (the same day)	Death

Abbreviations ANC: absolute neutrophil count; AML: acute myeloid leukemia; BDG: beta-D-glucan; CAR-T: Chimeric Antigen Receptor T-cell therapies; CNS: central nervous system; CR-BSI: catheter related bloodstream infection; F: female; GM: galactomannan; GvHD: Graft versus Host Disease; HCT: hematopoietic cell transplantation; L-Amb: liposomal amphotericin B; M: male; MM: multiple myeloma; MUD: matched unrelated donor; NA: non-available; NHL: non-Hodgkin lymphoma.

Table S4. Available antifungal Minimum Inhibitory Concentration of *M. clavatus* and *M. capitatus* in patients with IFI, 2022 and 2023

	MIC (mg/L)						
**Antifungal agent**	***M. clavatus* (HSM-S7)** (blood)	***M. clavatus* (HSM-S8)** (blood)	***M. clavatus*, (HSM-S9)** (blood, first episode)*	***M. clavatus*, (HSM-S9)** (blood, second episode)*	***M. clavatus* (HSM-S5)** (blood)	***M. capitatus* (HSM-S5)** (blood)	***M. capitatus* (HSM-S4)** (BALF)
**Amphotericin B**	1	1	0.5	0.250	0.5	0.5	2
**Fluconazole**	4	4	8	8	4	8	8
**Isavuconazole**	0.015	0.015	0.25	0.25	0.06	0.25	1
**Itraconazole**	0.06	0.06	0.120	0.250	0.06	0.25	0.250
**Posaconazole**	0.25	0.25	0.5	0.5	0.12	0.5	0.500
**Voriconazole**	0.03	0.03	0.120	0.120	0.03	0.12	0.250
**Anidulafungin**	1	1	2	2	2	2	1
**Caspofungin**	>8	>8	8	8	>8	>8	>8
**Micafungin**	1	1	2	2	>8	>8	>8
**Flucytosine**	NA	NA	NA	NA	NA	NA	NA

Abbreviation: IFI: invasive fungal diseases; MIC: Minimum Inhibitory Concentration; * isolated from the same patient after blood culture negativization

FIGURES

Figure S1. Magnetic Resonance Imaging of CNS disease due to *M. capitatus*

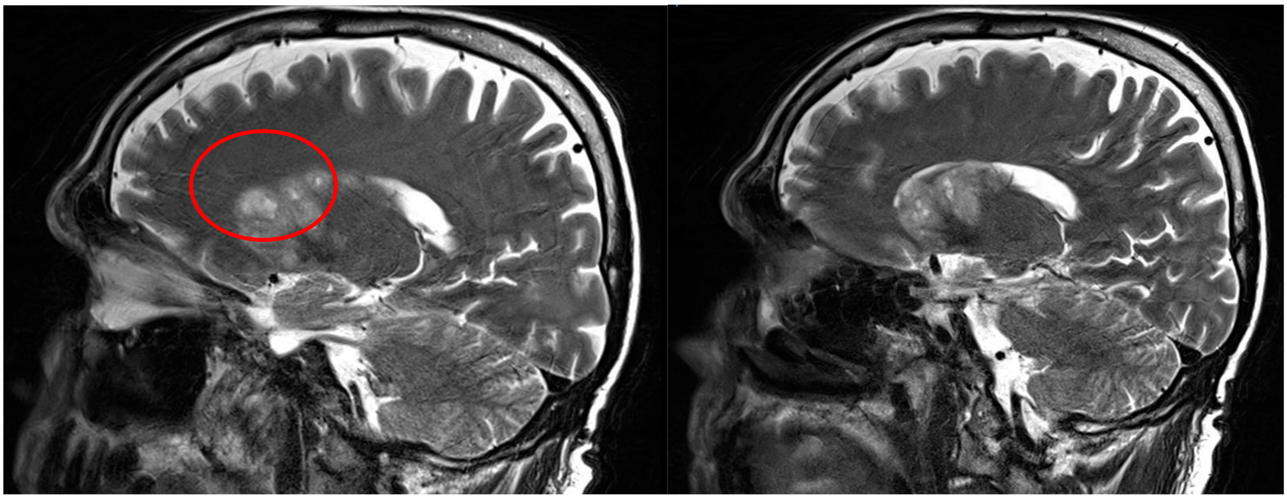

Description: extensive signal alteration with multiple nodular components, affecting the caudate nucleus and the lenticular nucleus bilaterally, with greater extension and tumefactive aspects at the pallidal and putaminal level on the right side.

Figure S2. Lung CT Imaging of pulmonary disease due to *M. capitatus*

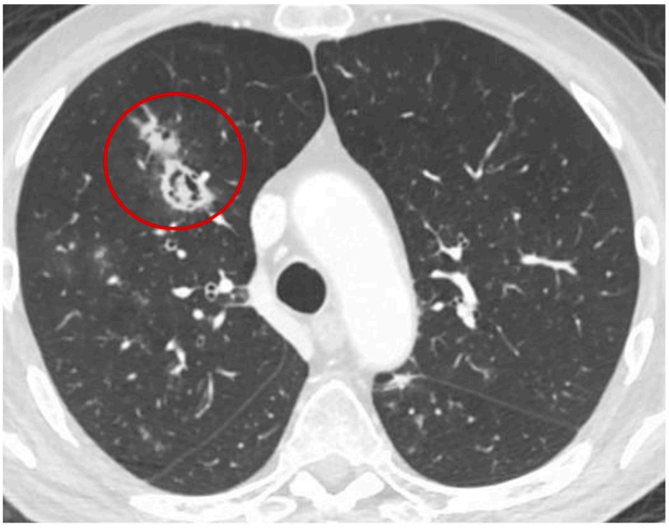

Description: pneumonia with excavated parenchymal peribronchovasal lesion localized at superior right lobe.

Figure S3. *Geotrichum* spp. and *Magnusiomyces* spp. strains isolated in our hospital from 2007 to 2023 with regular screening and routine microbiological tests

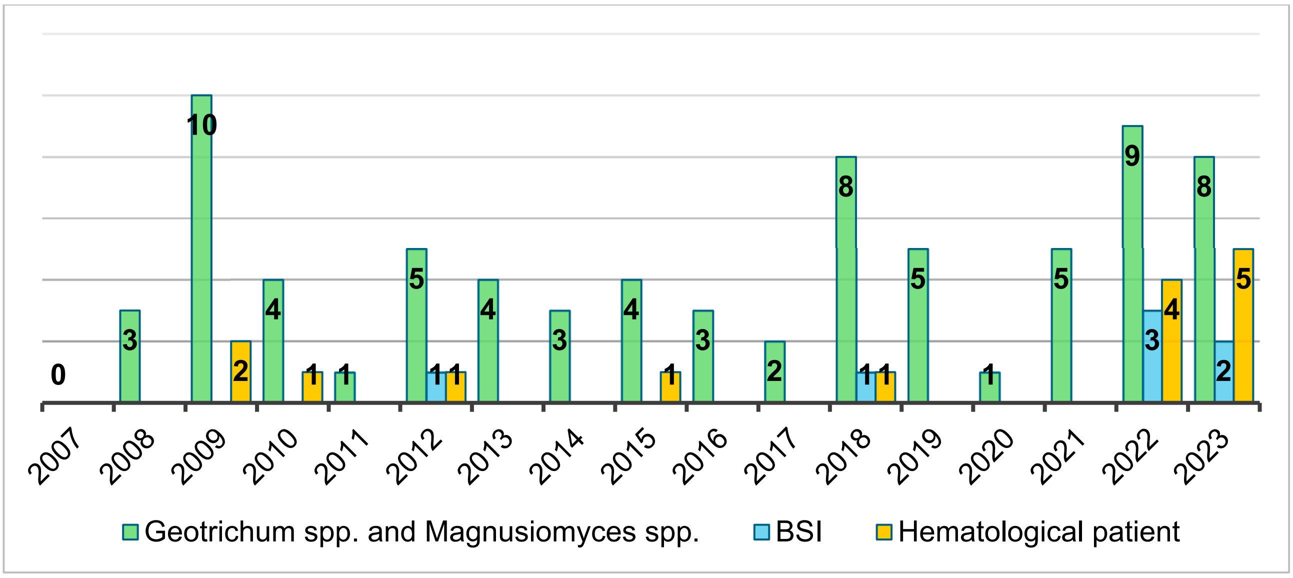

Abbreviations: BSI: bloodstream infection

Figure S4. Hepatosplenic invasive fungal disease due to *M. clavatus*

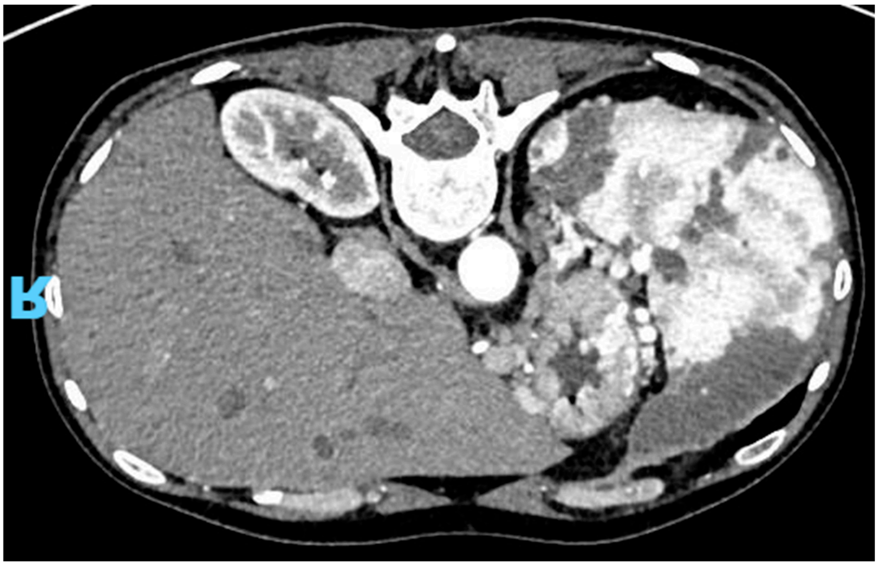

Supplement: Supplementary file 1 [file jof-12-00254-s001.zip › jof-4164964-supplementary.pdf]
